# Thermally and Photothermally Triggered Cytocompatible Triple-Shape-Memory Polymer Based on a Graphene Oxide-Containing Poly(ε-caprolactone) and Acrylate Composite

**DOI:** 10.1021/acsami.3c13584

**Published:** 2023-10-30

**Authors:** Junjiang Chen, Shiyang Sun, Mark M. Macios, Elizabeth Oguntade, Ameya R. Narkar, Patrick T. Mather, James H. Henderson

**Affiliations:** † BioInspired Syracuse: Institute for Material and Living Systems, Syracuse University, Syracuse, New York 13244, United States; ‡ Department of Biomedical and Chemical Engineering, Syracuse University, Syracuse, New York 13244, United States; § Department of Chemical Engineering, Penn State University, University Park, Pennsylvania 16802, United States

**Keywords:** triple-shape-memory polymers, thermal responsive, light responsive, cytocompatible, smart materials, functional materials

## Abstract

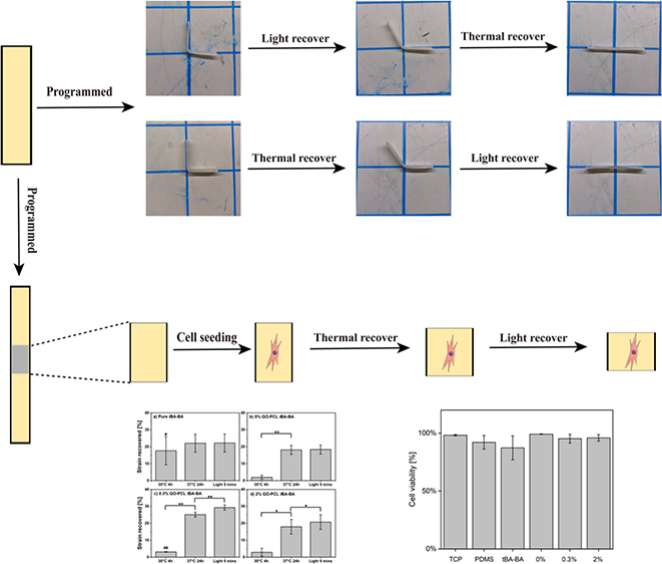

Triple-shape-memory polymers (triple-SMPs) are a class of polymers capable of fixing two temporary shapes and recovering sequentially from the first temporary shape to the second temporary shape and, last, to the permanent shape. To accomplish a sequential shape change, a triple-SMP must have two separate shape-fixing mechanisms triggerable by distinct stimuli. Despite the biomedical potential of triple-SMPs, a triple-SMP that with cells present can undergo two different shape changes via two distinct cytocompatible triggers has not previously been demonstrated. Here, we report the design and characterization of a cytocompatible triple-SMP material that responds separately to thermal and light triggers to undergo two distinct shape changes under cytocompatible conditions. Tandem triggering was achieved via a photothermally triggered component, comprising poly(ε-caprolactone) (PCL) fibers with graphene oxide (GO) particles physically attached, embedded in a thermally triggered component, comprising a *tert*-butyl acrylate-butyl acrylate (*t*BA-BA) matrix. The material was characterized in terms of thermal properties, surface morphology, shape-memory performance, and cytocompatibility during shape change. Collectively, the results demonstrate cytocompatible triple-shape behavior with a relatively larger thermal shape change (an average of 20.4 ± 4.2% strain recovered for all PCL-containing groups) followed by a smaller photothermal shape change (an average of 3.5 ± 0.8% strain recovered for all PCL-GO-containing groups; samples without GO showed no recovery) with greater than 95% cell viability on the triple-SMP materials, establishing the feasibility of triple-shape memory to be incorporated into biomedical devices and strategies.

## 1 Introduction

Shape memory polymers (SMPs) are a class of smart materials that, in their most basic one-way instantiation, can be deformed into a temporary shape and later recover to a permanent shape in response to an external stimulus.^1−4^ This one-way shape memory effect requires the presence of two polymeric functionalities: a shape recovery component that has a relatively higher transition temperature, is able to prevent flow deformation, and can maintain and recover to the original shape; and a shape-fixing component that has a relatively lower transition temperature, can undergo reversible changes, and is mobilized in response to an external stimulus.^5,6^ The temporary shape is obtained by mechanically deforming and immobilizing the SMP, for example, by heating above the material’s glass transition or melting temperature, deforming, cooling below the transition temperature to fix the temporary shape, and unloading. The temporary shape can be maintained until a stimulus is applied, at which point the material is triggered to recover to the permanent shape via the release of the energy of deformation that has been stored during shape fixing. Such stimuli include heat, light, solvent, ultrasound, and enzymatic activity.^7−9^

There has been substantial interest in developing cytocompatible and biocompatible SMPs. This interest has been motivated in large part by the potential of SMPs to enable the development of novel biomedical applications, both in benchtop research and in translation to clinical strategies in areas such as tissue engineering.^10^ For example, micro- or nano-sized SMPs are being studied to enable new strategies for targeted drug delivery,^11^ and macroscopic SMPs are being studied to enable new strategies for the treatment of orthopedic injury or disease.^12,13^

Triple-shape-memory polymers (triple-SMPs) are a class of polymers capable of fixing two temporary shapes and recovering sequentially from the first temporary shape to the second temporary shape and, last, to the permanent shape. To possess triple-shape behavior, an SMP must have two separate shape-fixing mechanisms triggerable by distinct stimuli.^14−18^ In the case of sequential thermal triggers, the temperatures at which triggering occurs must be sufficiently separated so as to decouple the two recovery steps while conversely being close enough together that the temperature range experienced by the SMP and its surroundings is compatible with the desired application.

Triple-SMPs have the potential to meet previously unaddressed needs in biomedical application areas that include controlled release for drug delivery^19^ and smart scaffolds.^20^ For example, in drug delivery, a triple shape could provide the stepwise release of a single payload to a target or, alternatively, sequential release of multiple payloads to different targets to avoid damage to untargeted tissues. With smart scaffolds, a triple shape could allow for fixation in a compact conformation for minimally invasive delivery and deployment to the working conformation (shape change 1) followed by an activation of the therapeutic shape change at the desired time of intervention (shape change 2). Despite the biomedical potential of triple-SMPs, research on cytocompatible SMPs has, to date, been limited almost exclusively to single-step one-way SMPs^21−25^ with some work on cytocompatible two-way SMPs,^26,27^ which can reversibly switch between two shapes.^27,28^ There has been only one prior report on a triple-SMP that can under model *in vitro* conditions be triggered at body temperature and that showed no cell toxicity in an indirect (media elution) assay, but shape-memory triggering was not studied with cells attached to the material or otherwise present.^29^ As such, a cytocompatible triple-SMP that with cells present can undergo two different shape changes via two distinct cytocompatible triggers has not previously been demonstrated.

The major challenge to developing a cytocompatible triple-shape material has been that thermal triggering—the most widely studied form of shape-memory triggering—must for a triple shape include trigger temperatures far enough apart to allow two different shape changes but close enough together for both temperatures to remain within a cytocompatible range,^30^ neither dropping significantly below nor, more importantly, rising significantly above body temperature, particularly for an extended duration. One potential solution is to trigger one or both shape changes in an indirect manner,^7^ i.e., one that does not involve an increase in ambient temperature, for example, via photothermal triggering of the SMP material.^31^ In addition to providing a mechanism whereby heating would be largely or completely restricted to the material with a minimal temperature change to its surrounding, light-based stimulation provides an opportunity both for spatial control of the region triggered^31^ and for noncontact activation that can penetrate surrounding matter, such as culture medium or tissue, with minimal heating thereof.^32^ Established dopants capable of imparting photothermal properties to materials include gold nanorods and nanoparticles,^33^ graphene oxide,^34,35^ and carbon nanomaterials.^36^

Motivated by the potential for triple-SMPs to enable new biomedical strategies and devices, we report the design and characterization of a cytocompatible triple-SMP that responds to both a thermal trigger and a light trigger to undergo two distinct shape changes under cytocompatible conditions. Tandem triggering was achieved via a photothermally triggered component, comprising poly(ε-caprolactone) (PCL) fibers with graphene oxide particles physically attached, embedded in a thermally triggered component, comprising a *tert*-butyl acrylate-butyl acrylate (*t*BA-BA) matrix. The material was characterized in terms of thermal properties, surface morphology, shape-memory performance, and cytocompatibility following triggering and a shape change.

## 2 Methods

### 2.1 Triple-Shape Scheme and Study Design

**Scheme 1:**
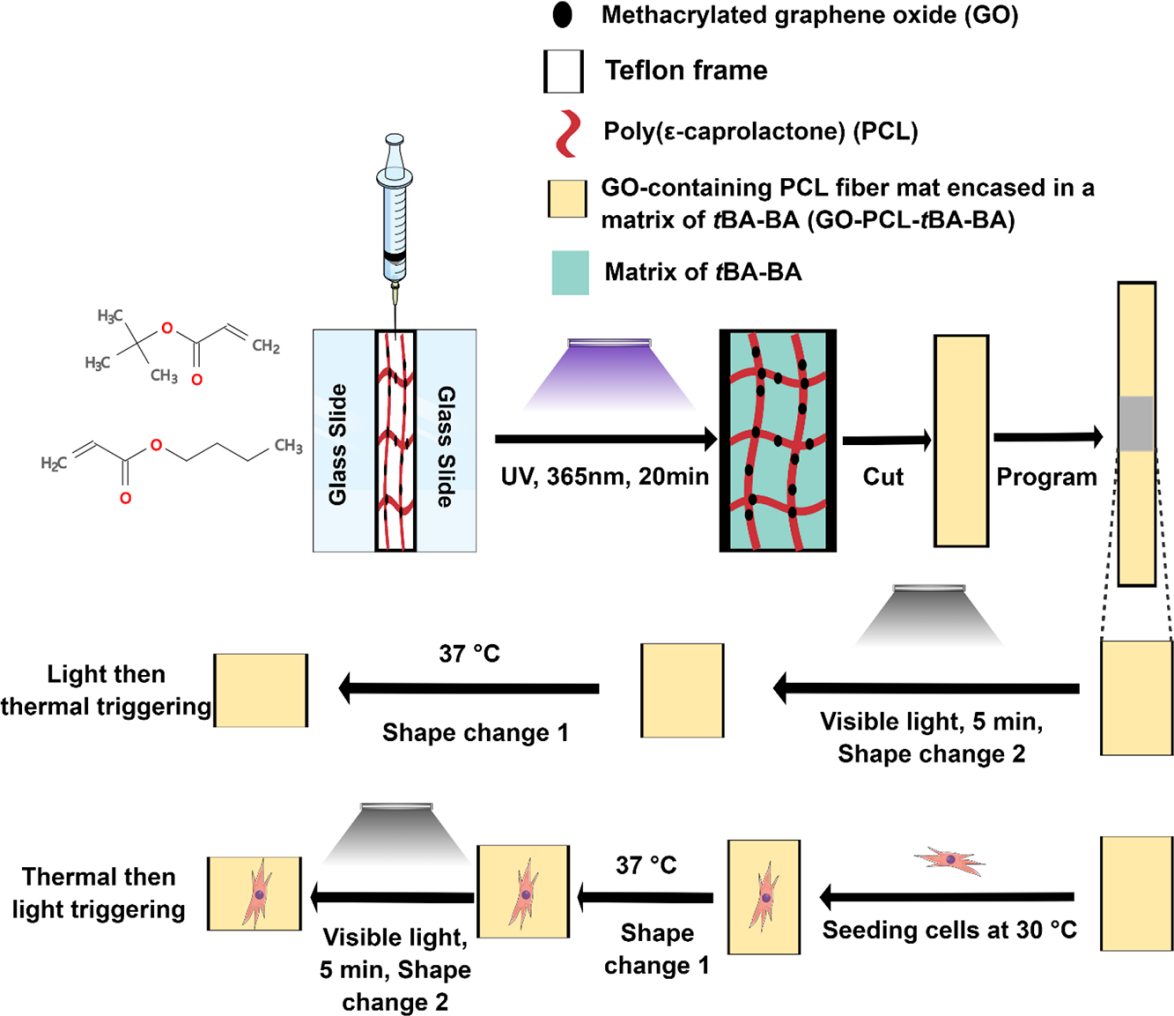
Cytocompatible Triple-SMP Fabricated by Encasing a GO-Containing PCL Fiber Mat in a Matrix of *t*BA-BA SMP samples were composed of two parts: *t*BA-BA copolymer comprised the thermally triggered component (shape change 1), and PCL fibers with GO particles physically attached comprised the photothermally triggered component (shape change 2). *t*BA-BA (*tert*-butyl acrylate-butyl acrylate), GO (methacrylated graphene oxide), and PCL (poly(ε-caprolactone)).

The cytocompatible triple-shape design scheme (Scheme 1 and Scheme S1) was to fabricate a composite SMP with a methacrylated graphene oxide (GO)-containing PCL fiber mat encased in a matrix of *t*BA-BA. SMP samples were composed of two parts: *t*BA-BA copolymer comprised the thermally triggered component (shape change 1); and PCL fibers with GO particles physically attached comprised the photothermally triggered component (shape change 2). Both possible orders of triggering were studied—thermal triggering followed by photothermal triggering and vice versa. Regardless of the order in which triggering was being studied, thermal triggering was performed by heating a sample to 37 °C, a temperature low enough to avoid melting PCL fibers but with shape recovery (shape change 1) achieved via melting of the *t*BA-BA copolymer. Photothermal triggering was performed by exposure to light, with shape recovery (shape change 2) achieved via the photothermal effect, as GO trapped in PCL fiber mats caused the PCL to melt.

Once fabricated, the thermal properties of the SMP were analyzed by thermogravimetric analysis and differential scanning calorimetry, the surface morphology of PCL fibers was analyzed by scanning electron microscopy, and shape-memory performance was analyzed by dynamic mechanical analysis. Cytocompatibility was analyzed by the Live/Dead assay with C3H10T1/2 cells cultured on the SMP during triggering and shape change. These methods are described in more detail in the following sections.

### 2.2 Materials

*tert*-Butyl acrylate (*t*BA), butyl acrylate (BA), unexfoliated GO, PCL (Mn = 80,000 g/mol) pellets, 2,2-dimethoxy-2-phenylacetophenone (DMPA), tetraethylene glycol dimethacrylate (TEGDMA), *N*,*N*-dimethylformamide (DMF), chloroform (CHCl_3_), and methanol were purchased from Sigma-Aldrich. SDHR-4 purification columns (Scientific Polymer) were used to remove the inhibitors in *t*BA and BA. C3H10T1/2 mouse embryonic fibroblast cells were obtained from the American Type Culture Collection (ATCC) used at passages 13–15 for experiments. l-Glutamine solution was purchased from ATCC. Fetal bovine serum (FBS, LOT 1119174), Dulbecco′s phosphate-buffered saline (PBS), penicillin/streptomycin (P/S), and Basal Medium Eagle (BME) were purchased from Gibco and used as received. The Live/Dead assay was obtained from Invitrogen and used as received.

### 2.3 Methacrylated GO Preparation

Unexfoliated GO paste was exfoliated following the manufacturer’s procedure. Briefly, 1 g of GO paste was mixed with 25 mL of water with continuous stirring for 16 h, followed by sonication for 30 min. The mixture was then centrifuged at 2460 × *g* for 10 min to remove the unexfoliated GO. The supernatant was dried via a rotavapor and then in a vacuum oven and resuspended in ethanol to yield a 1 mg/mL solution. Following the method established by Cha et al.,^37^ 50 μL of 3-(trimethyloxysilyl)propyl methacrylate per mg GO was slowly added to the solution with continuous stirring, and the solution was sonicated for 60 min, followed by stirring at 50 °C for 12 h. The resultant mixture was dialyzed (Spectrum, 12 to 14 kDa) against aqueous ethanol and dried in the vacuum oven to obtain the methacrylated GO. To confirm the successful modification of GO with polymerizable acrylate groups, Fourier transform infrared (FTIR) spectroscopy was performed (Figure S1).

### 2.4 PCL Fabrication

PCL particles were dissolved in CHCl_3_ and DMF (CHCl_3_:DMF = 4:1 v/v%). GO was added to the PCL solution with different weight percentages studied (0% GO-PCL, 0.3% GO-PCL, and 2% GO-PCL). GO-PCL (0%) (pure PCL) was used as a control, as it should exhibit negligible (baseline) photothermal behavior. Solutions were stirred overnight until the GO was homogeneously dispersed in the PCL solution. GO-PCL solution was electrospun to form fiber mats. The custom electrospinning apparatus used includes a rotating cylindrical drum collector (5 cm diameter), an electrospinning syringe pump (Thermo Fisher), a high-voltage positive power supply (Agilent E3630A), and a low-voltage negative power supply (PS 500XT, Hoefer Scientific). A negative voltage of −500 V was applied to the mandrel to improve the fiber deposition. The flow rate of the solution was set at 2 mL/h, with a total delivered volume of 20 mL. Voltages (12 kV) were applied to the 22 G needle tip, with a needle-to-mandrel distance of 10 cm and a rotation speed of 400 rpm. Using ImageJ, scanning electron micrographs (Section 2.6) were analyzed to determine the diameter of PCL fibers and the distribution density of GO particles in the different material compositions (Table S1).

### 2.5 Preparation of PCL Fiber Mats Embedded in a Polymerized Network

Following our established method,^38−40^ a weight percent ratio of *t*BA:BA of 95:5 was selected to provide a *t*BA-BA composition that would recover at approximately 37 °C. Additionally, to the total weight of the *t*BA and BA mixture, 0.1 wt % DMPA (photoinitiator) and 5 wt % TEGDMA (cross-linker) were added. To encase the PCL fiber mat in the *t*BA-BA matrix, we first fixed the GO-PCL fiber mats in a frame comprising two Rain-X-treated glass slides with a 1 mm Teflon spacer. *t*BA-BA solution was then injected into the frame to saturate the PCL fiber mats. Pure *t*BA-BA solution with no GO-PCL fiber mat was used as a control that should be unresponsive to light triggering. After injection, all samples were placed in a UV curing chamber (>300 J/cm^2^, CL-3000L, 365 nm, Analytik Jena) and cured for 20 min. The resulting polymer composites were immersed in 50% methanol/deionized water solution overnight to remove the unreacted monomers and then dried in a vacuum oven at room temperature for 72 h.

### 2.6 Imaging of GO-PCL Fibers

Vacuum-dried GO-PCL samples obtained from polymer films recovered by either light or heat were coated with Au for 45 s (Denton Vacuum-Desk II) and characterized by using a scanning electron microscope (SEM, JEOL 5600) with an accelerating voltage of 10 kV.

### 2.7 Quantification of Thermal Properties

Thermogravimetric analysis (TGA, TA Instruments Q500) was used to examine the thermal degradation behavior of the prepared SMPs. Samples were heated to 600 °C at a maximum rate of 10 °C/min. As previously described,^8^ a resolution of 4 °C and a sensitivity value of 1 (instrument model specific) were used.

Differential scanning calorimetry (DSC, TA Instruments Q200 with a liquid nitrogen cooling system) was used to determine the crystallinity of the samples. During each experiment, samples weighing between 3 and 5 mg were loaded into a “premium pan” (DSC Consumables) and equilibrated by cooling to −30 °C. Samples were then heated to 100 °C and cooled to −30 °C at 10 °C/min to remove any thermal history present in the sample. Samples were then subjected to heating at 10 °C/min to 100 °C and the glass transition (*T*_g_) determined from the inflection point during the transition in the heating curve using Universal Analysis software (TA Instruments).

The PCL composition of each sample was estimated by the heat of crystallization via eq 1, where Δ*H*_pcl-comp_ is the melting enthalpy of PCL in the GO-PCL-*t*BA-BA polymer composites and Δ*H*_pcl-pure_ is the melting enthalpy of pure PCL:
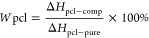
1

### 2.8 Shape Memory Performance

#### 2.8.1 Analysis of Thermal Triggering

Shape memory cycles with thermal triggering were carried out in a force-control mode on a dynamic mechanical analyzer (DMA, TA Instruments Q800). Each sample was heated to 55 °C and loaded at 0.05 N/min until 20% strain was achieved. The sample was next cooled at 2 °C/min to 0 °C, followed by releasing the applied force at 0.1 N/min. Finally, the sample was reheated at 2 °C/min to 55 °C to complete one cycle. The cycle was repeated three times. The fixing (*R*_f_) and thermally triggered recovery (*R*_r,th_) ratios were calculated each cycle from eqs 2 and 3, where ε_*x*_ and ε_*x,*load_ are, respectively, the strain fixed and the targeted programmed strain, ε_*y*_ is the strain (relative to the start of the cycle) remaining after thermal recovery, and ε_*y*,res_ is the residual strain from the previous cycle:
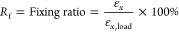
2
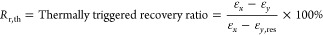
3

The fixing and recovery ratios measure how well the material maintains the fixed shape and recovers the permanent shape, respectively, with 100% being perfect fixing and recovery.^41^

#### 2.8.2 Analysis of Light Triggering

Shape memory cycles with light triggering were separately characterized using the DMA. First, a sample was loaded into the DMA and heated to 55 °C, a temperature above the *T*_g_ of *t*BA-BA and melting transition (*T*_m_) of PCL. The sample was then stretched at a rate of 0.08 N/min until a strain of 20% was reached. The sample was then cooled to 0 °C at a rate of 3 °C/min and held isothermally for 5 min, and the force was then unloaded at a rate of 0.1 N/min. At this point, the furnace was opened, and the DMA was programmed to isothermally hold the temperature for 30 min. The opening of the oven prevented the temperature controller from activating and allowed the DMA to record data, as it normally does, while the sample was exposed to light. The sample was exposed to visible light with an intensity of 8.69 mW/mm^2^ using a high-power wide-spectrum light source (Fiber-Lite DC-950, Dolan-Jenner Industries; kindly provided by the Hosein Lab at Syracuse University) with a flexible gooseneck made from optical fibers with a steel cladding used as the light guide. The light guide prevented any heat from the halogen bulb from affecting the sample, thus ensuring that any observed sample recovery was due solely to light exposure. Following the 30 min of light exposure, the light-triggered recovery ratio (*R*_r,L_) was calculated as the ratio of the recovered strain to the programmed strain using eq 4:
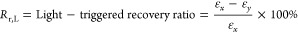
4

ε_l_ is the strain achieved after light recovery. The fixing ratio (*R*_f_) was again calculated from eq 2.

#### 2.8.3 Qualitative and Quantitative Demonstration of Thermal- and Light-Triggered Recovery

To visualize the triple-shape recovery of the SMPs, the samples were programmed into a temporary shape by heating at 55 °C for 5 min and then manually deforming by bending to a 90° angle in a bracket mold (Supplemental 1 and Figure S3). The samples were then cooled down in a refrigerator at −20 °C and then separated into two treatment groups representing the two orders of triggering: (1) heat at 37 °C in PBS for 24 h, followed by exposure to 8.69 mW/mm^2^ visible light for 5 min; or (2) exposure to 8.69 mW/mm^2^ visible light for 5 min, followed by heating at 37 °C in PBS for 24 h. The temperature of the material after being exposed to visible light with an intensity of 8.69 mW/mm^2^ for 5 min was recorded by an IR camera (Table S4). The bending angle was measured using ImageJ. The fixing ratio was calculated from eq 5 and the recovery ratio was calculated from eq 6, where θ_*x*_ and θ_*x,*load_ are, respectively, the angle fixed and the targeted programmed angle and θ_*y*_ is the angle remaining after recovery:
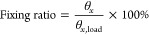
5
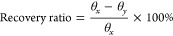
6

### 2.9 Cytocompatibility and Shape Change during Cell Culture

To study the shape changes and cytocompatibility of both thermal and light triggering under cell culture conditions, experiments were conducted using the C3H10T1/2 cell line, which we have used extensively when characterizing cytocompatible SMPs.^8,42^ To minimize the duration for which cells would be cultured at 30 °C—a cytocompatible but nonoptimal culture temperature below the thermal trigger temperature—the triggering order of thermal triggering followed by light triggering was chosen. A complete cell culture medium was prepared with BME, 10% FBS, 1% l-glutamine, and 1% penicillin/streptomycin (P/S). Freshly prepared GO-PCL-*t*BA-BA samples were cut into 10 mm × 5 mm rectangles, and by using a custom screw-driven manual stretcher, the samples were stretched to 20% strain under 55 °C and fixed at −20 °C. The programmed SMPs were then cut into 5 mm × 5 mm pieces for cell culture. The samples were UV-sterilized for a minimum of 24 h in a UV sterilizer (SkinAct) and then soaked in complete BME overnight under room temperature to allow protein absorption to the surface before cell culture. As the samples would float in the medium if not anchored due to their low density, polydimethylsiloxane (PDMS) was used to coat the bottom of a 24-well tissue culture plate and affix the samples to the tacky PDMS. Cells were seeded on the samples with a seeding density of 10^4^ cells/well, with a total medium volume of 0.5 mL/well, and incubated in a 30 °C incubator for 4 h to allow cell attachment to the samples. The samples were then transferred to a 37 °C incubator and cultured for an additional 20 h, during which time the first (thermal) shape recovery event was triggered by recovery of *t*BA-BA, while PCL remained unmelted. Sample recovery occurred within the first ∼2 h of culture at 37 °C, with the remainder of the 20 h culture conducted to permit any deleterious effects to manifest, were they going to occur. The samples were then exposed to visible light with an intensity of 8.69 mW/mm^2^ for 5 min, triggering a second recovery event. The sample length was measured by a caliper in the biosafety cabinet before recovery and then following 30 °C incubation, 37 °C incubation, and light recovery, respectively. The strain recovered was calculated as the ratio of the change in length to the original length. Following the triggering events, the Live/Dead assay was used to analyze cell viability. Live cells were labeled by green-fluorescent calcein-AM, which detects intracellular esterase activity. Dead cells were labeled by red-fluorescent ethidium homodimer-1 (EthD-1), which detects the loss of plasma membrane integrity. Cells on tissue culture well plates without samples present were used as negative (nontoxic) controls, and cells on PDMS in the absence of samples were used as an additional control for any effect of PDMS. Twenty minutes before staining, the positive dead (toxic) control was prepared by removing the cell culture medium, washing with PBS, and adding 70% ethanol. Cell viability was determined as the percentage of live stained cells (those not presenting dead stain) among the total cells counted.

### 2.10 Statistical Methods

All experiments were repeated three times. Multiple comparisons were made by one-way ANOVA followed by Tukey’s post hoc test between groups. Significances were set at *p* < 0.05. The results are reported as mean ± standard deviation.

## 3 Results

### 3.1 SEM Imaging of GO-PCL Fibers

**Figure 1:**
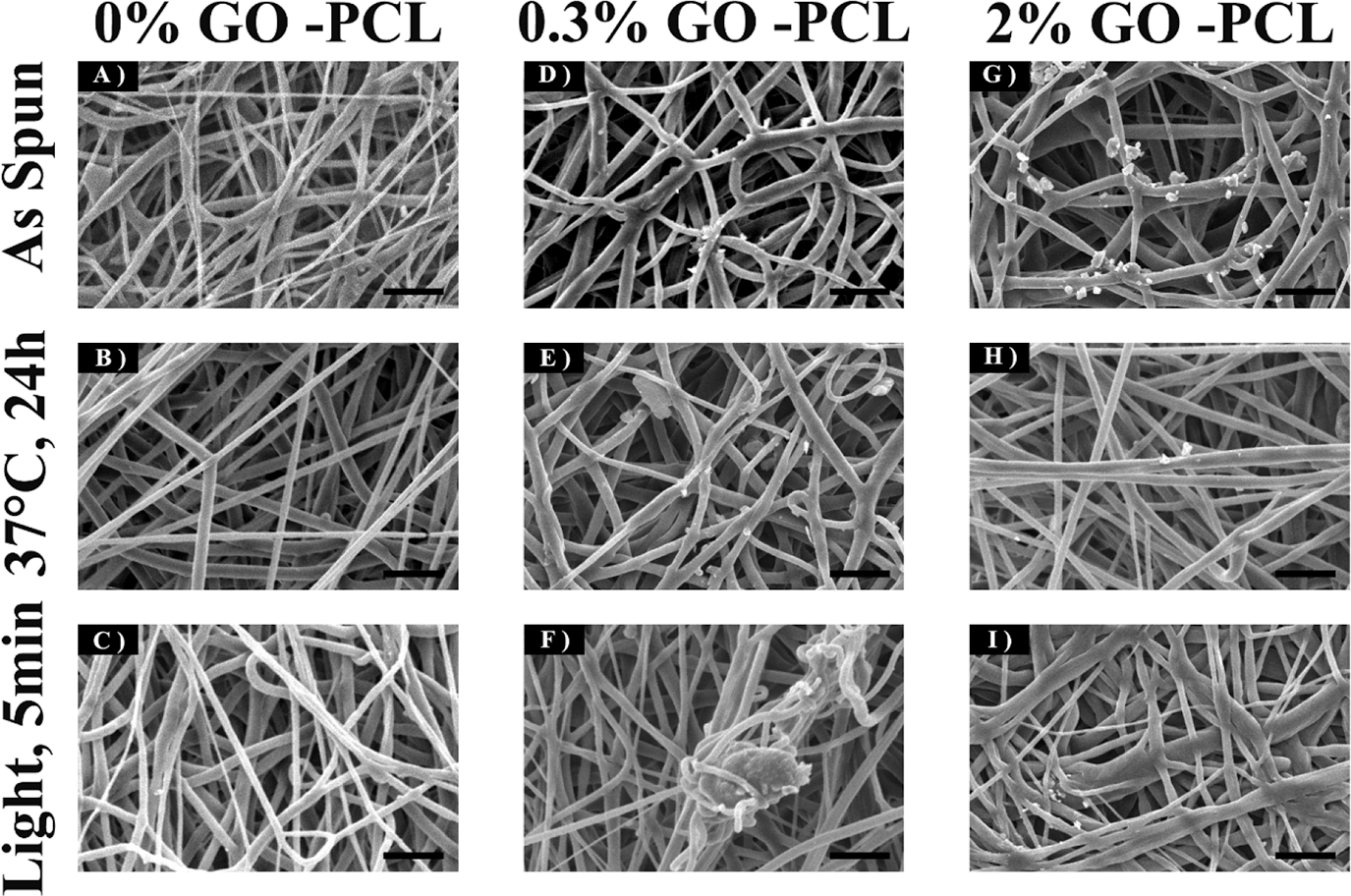
SEM imaging revealing that the presence of GO led to the melting of the fiber structure when exposed to visible light with an intensity of 8.69 mW/mm^2^ for 5 min. (A–C) As-spun, heat-treated, and light-treated PCL fibers with 0% GO. (D–F) As-spun, heat-treated, and light-treated PCL fibers with 0.3% GO. (G–I) As-spun, heat-treated, and light-treated PCL fibers with 2% GO. GO (methacrylated graphene oxide) and PCL (poly(ε-caprolactone)). Scale bar: 10 μm.

SEM imaging revealed that the presence of GO led to the melting of the PCL fiber structure upon exposure to visible light with an intensity of 8.69 mW/mm^2^ for 5 min (Figure 1). In contrast, no samples showed morphological changes after heating to 37 °C in PBS for 24h. Similarly, PCL fibers with 0% GO showed no melting-related change in fiber morphology after the completion of both the heat and light triggering events (Figure 1C). Coarse fibers were observed in both experimental groups containing GO, which suggests the melting of PCL (Figure 1F,I). We would not expect such coarsening of fibers to be observed if a matrix was present.

### 3.2 Thermal Properties

**Figure 2:**
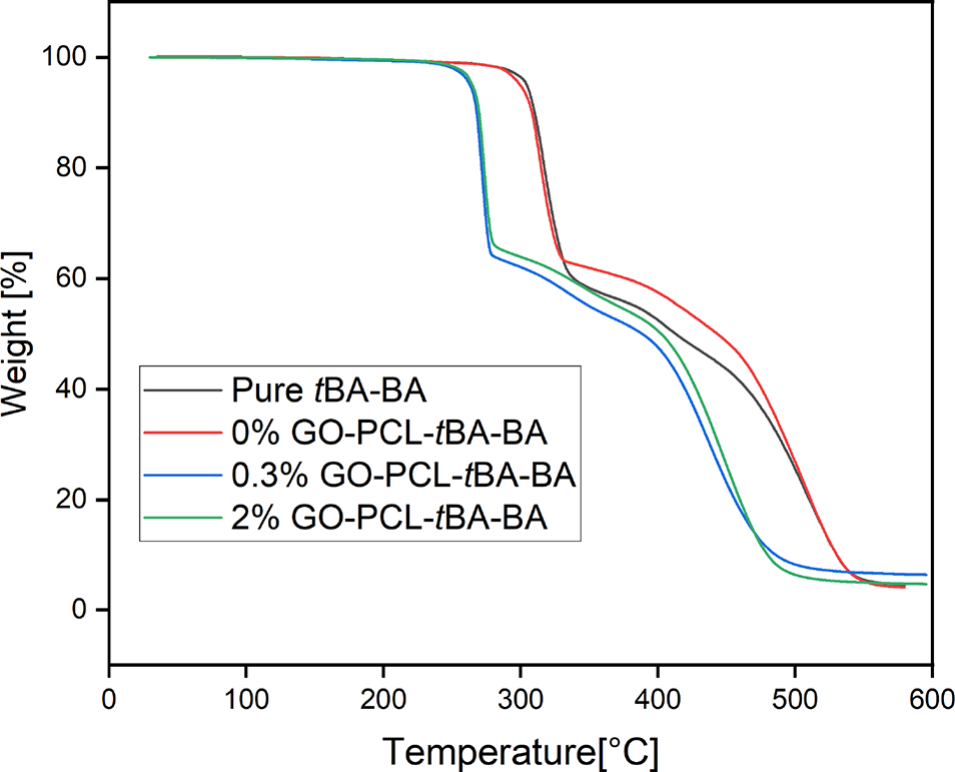
Representative traces from TGA showing a two-step degradation of all samples, which is consistent with the anticipated degradation of *t*BA-BA. Samples with a GO component show a first degradation that starts from a lower temperature than was observed in samples without a GO component. *t*BA-BA (*tert*-butyl acrylate-butyl acrylate), GO (methacrylated graphene oxide), and PCL (poly(ε-caprolactone)).

TGA showed a two-step degradation of all samples, which is consistent with the anticipated degradation of *t*BA-BA (Figure 2). A first degradation of samples without GO occurred in the range from 280 to 350 °C, with a 40% weight loss and a maximum decomposition rate occurring at 315 °C, followed by a second degradation ending at 555 °C, with an additional 55% weight loss and a maximum decomposition rate occurring at 510 °C. In contrast, the samples with GO showed a first degradation that started from a lower temperature of 240 °C, followed by a second degradation ending at 510 °C, with 35 and 60% weight losses and maximum decomposition rates occurring at 270 and 450 °C, respectively. It should be noted that the degradation of the GO-containing samples occurred at a lower temperature than no GO samples, which suggested a decease in the thermal stability of the polymer composites with the addition of GO. This thermal stability decrease might be related to the loss of labile oxygen GO groups^43^.

**Figure 3:**
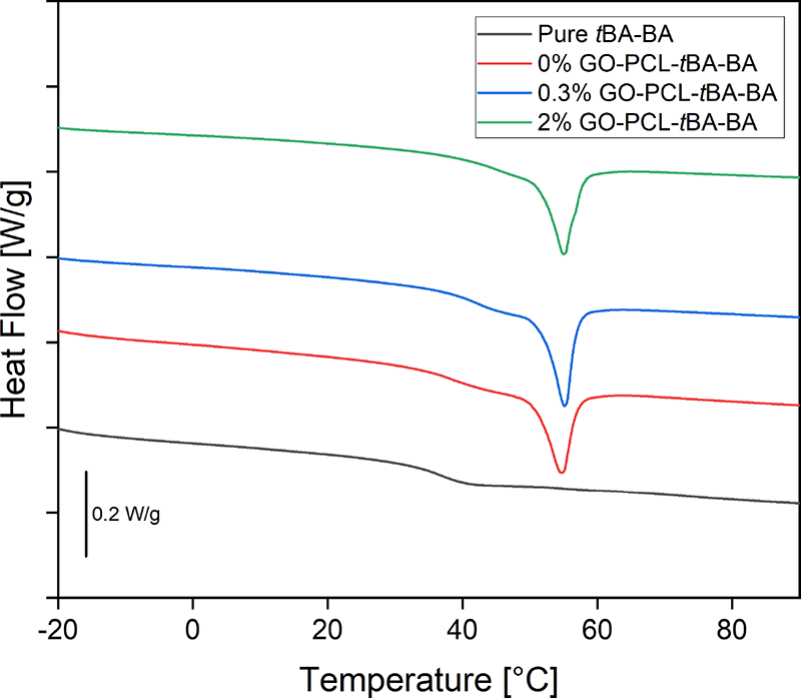
Representative traces from DSC analysis showing a glass transition (*T*_g_) of *t*BA-BA of approximately 36 °C and a melting transition (*T*_m_) of PCL of approximately 56 °C. All PCL-embedded samples showed a PCL weight percentage of 8.5 ± 0.3%. *t*BA-BA (*tert*-butyl acrylate-butyl acrylate), GO (methacrylated graphene oxide), and PCL (poly(ε-caprolactone)).

DSC analysis showed a *T*_g_ of *t*BA-BA of approximately 36 °C and a *T*_m_ of PCL of approximately 56 °C (Table S2 and Figure 3), which are consistent with our prior experiences with similar compositions.^8,35^ All PCL-embedded samples showed a PCL weight percentage of 8.5 ± 0.3%.

### 3.3 Shape Memory Analysis

#### 3.3.1 Thermal-Triggered Recovery

**Figure 4:**
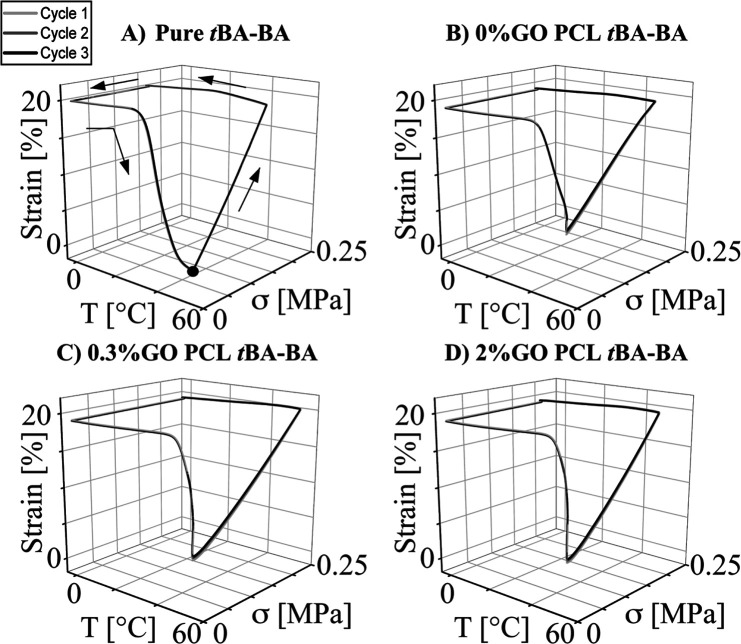
Representative traces from dynamic mechanical analysis (DMA) of thermal-triggered shape memory behavior showing strong shape-fixing and recovery properties for all samples. (A) pure *t*BA-BA, (B) *t*BA-BA with PCL fibers with no GO, (C) *t*BA-BA with PCL fibers with 0.3% GO, and (D) *t*BA-BA with PCL fibers with 2% GO. Black dot: starting point. *t*BA-BA (*tert*-butyl acrylate-butyl acrylate), GO (methacrylated graphene oxide), and PCL (poly(ε-caprolactone)).

Thermal shape memory cycles demonstrated strong shape fixing and recovery (Figure 4), with all sample compositions showing a fixing ratio (*R*_f_) over 97% and a thermally triggered recovery ratio (*R*_r,th_) over 85%.

#### 3.3.2 Light-Triggered Recovery

**Figure 5:**
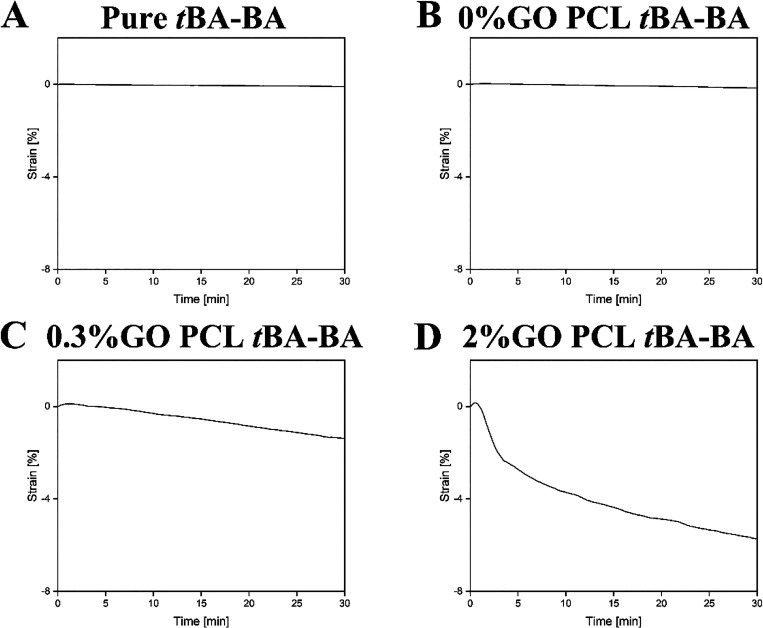
Representative traces from DMA showing no measurable shape recovery of samples with no GO (Figure 4A,B) but shape recovery with maximum strain changes of 1.5 and 6% of samples containing 0.3% GO (Figure 4C) and 2% GO (Figure 4D), respectively. (A) pure *t*BA-BA, (B) *t*BA-BA with PCL fibers with 0% GO, (C) *t*BA-BA with PCL fibers with 0.3% GO, and (D) *t*BA-BA with PCL fibers with 2% GO. *t*BA-BA (*tert*-butyl acrylate-butyl acrylate), GO (methacrylated graphene oxide), and PCL (poly(ε-caprolactone)).

DMA confirmed that all samples were successfully thermally programmed to 20% strain before light recovery (Figure S2). When these thermally programmed samples were exposed to intense visible light, DMA found that samples with no GO showed no measurable shape recovery (Figure 5A,B). In contrast and as intended, samples containing 0.3% GO (Figure 5C) and 2% GO (Figure 5D) showed maximum strain changes of 1.5 and 6%, respectively.

#### 3.3.3 Macroscopic Visualization of Triple-Shape Recovery

**Figure 6:**
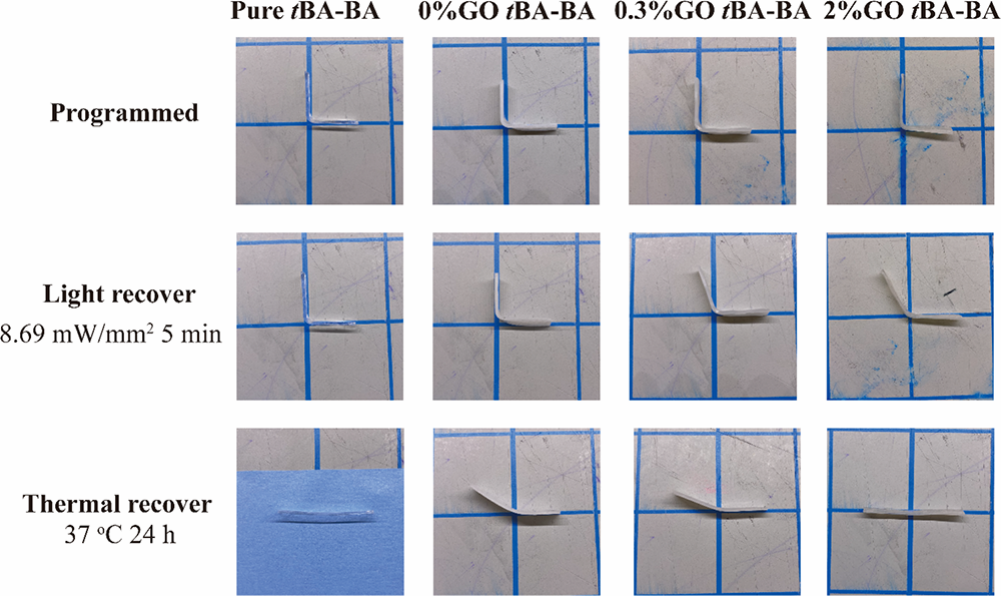
Representative photographs showing the stepwise shape recovery of samples after exposure to visible light followed by heating. The temporary shape was programmed by first heating the samples to 55 °C and then manually bending them to a 90° angle in a bracket mold and fixing them at −20 °C. For stepwise shape recovery, samples were first exposed to visible light with an intensity of 8.69 mW/mm^2^ for 5 min followed by heating at 37 °C in PBS for 24 h. *t*BA-BA (*tert*-butyl acrylate-butyl acrylate) and GO (methacrylated graphene oxide). Sample length: 60 ± 5 mm.

When manually bent to a 90° angle in a bracket mold, the stepwise shape recovery of the samples is visible macroscopically, whether heating is followed by exposure to visible light (Figure S4 and Table S3) or exposure to visible light is followed by heating (Figure 6 and Table 1). When samples were heated at 37 °C in PBS for 24 h followed by exposure to visible light for 5 min, all samples first, during thermal triggering, recovered from the 90° angle to a minimally bent shape and then, during light triggering, either recovered to completely flat (samples with GO; Figure S4) or showed no significant additional recovery (samples without GO). In contrast, when samples were exposed to visible light for 5 min followed by heating at 37 °C in PBS for 24 h, samples first, during light triggering, either started to show recovery from the programmed angle (samples with GO; Figure 6) or showed no significant recovery (samples without GO) and then, during thermal triggering, recovered to flat.

**Table 1:** Stepwise Shape Recovery after Exposure to Visible Light, Followed by Heating

exposure to visible light followed by heating	shape-fixing ratio	shape recovered after exposure to light	shape recovered after heating	total shape recovered
pure *t*BA-BA	97.55 ± 0.24%	1.13 ± 0.67%	91.66 ± 1.71%	92.79 ± 2.34%
0% GO-PCL- *t*BA-BA	95.70 ± 2.11%	2.95 ± 2.08%	67.96 ± 5.58%	70.91 ± 6.24%
0.3% GO-PCL- *t*BA-BA	98.11 ± 0.74%	12.81 ± 9.19%	55.51 ± 10.36%	68.32 ± 7.28%
2% GO-PCL- *t*BA-BA	98.27 ± 0.06%	28.99 ± 9.87%	51.40 ± 10.13%	80.39 ± 10.41%

### 3.4 Cytocompatibility and Shape Change during Active Cell Culture

**Figure 7:**
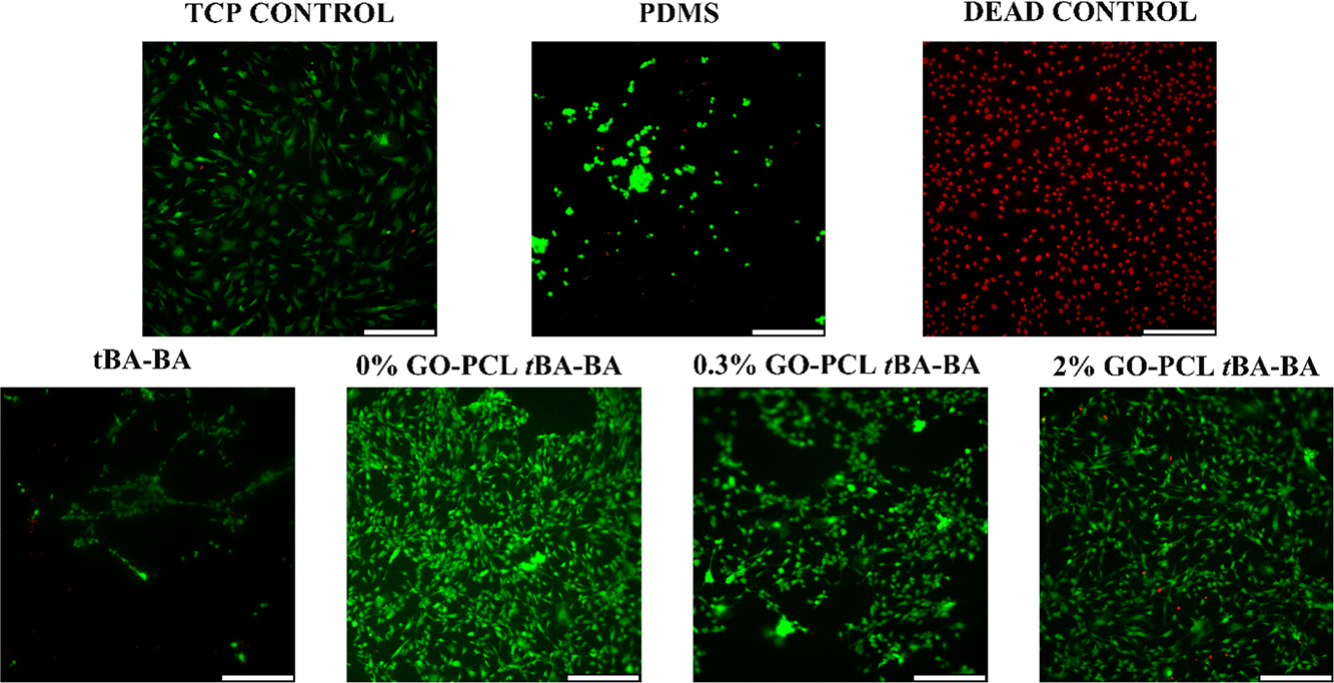
Live/Dead micrographs of C3H10T1/2 cells taken after triple-shape recovery showing high sample cytocompatibility. Few dead cells (red dots) were observed in any of the groups, except for the dead control. TCP (tissue culture plate), PDMS (polydimethylsiloxane), *t*BA-BA (*tert*-butyl acrylate-butyl acrylate), GO (methacrylated graphene oxide), and PCL (poly(ε-caprolactone)). Scale bar: 330 μm.

**Figure 8:**
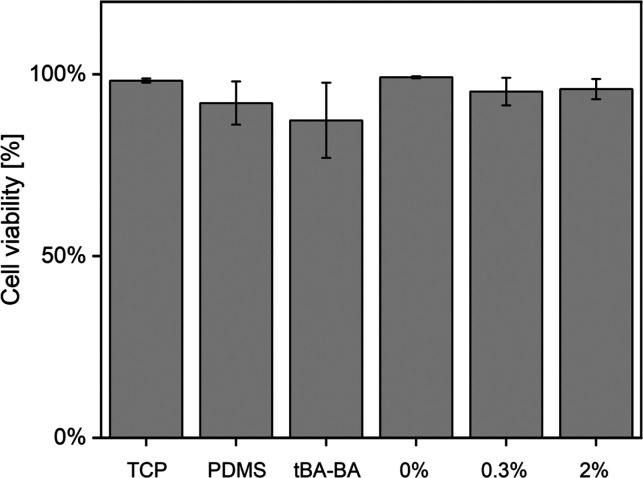
Live/Dead assay results showing high cytocompatibility when C3H10T1/2 cells are present on the samples during triple-shape recovery. All groups except pure *t*BA-BA and PDMS showed cell viability over 95%, while pure *t*BA-BA showed 87% cell viability and PDMS showed cell viability around 92%. 0, 0.3, and 2% represent 0, 0.3, and 2% GO-PCL-*t*BA-BA, respectively. TCP (tissue culture plate), PDMS (polydimethylsiloxane), *t*BA-BA (*tert*-butyl acrylate-butyl acrylate), GO (methacrylated graphene oxide), and PCL (poly(ε-caprolactone)).

**Figure 9:**
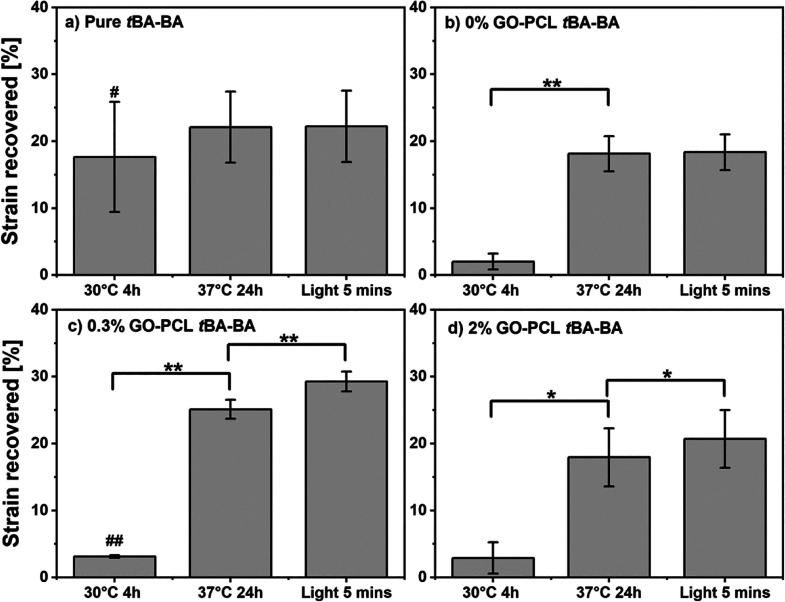
When samples were recovered via thermal triggering, followed by light triggering, successful triple-shape-memory behavior under cytocompatible conditions was demonstrated by the samples that contained GO, which showed statistically significant contractile length changes following both triggering events. In contrast, samples with no GO showed no significant change following the light triggering event. The strain recovered was calculated as the ratio of the sample length recovered to the original length. *t*BA-BA (*tert*-butyl acrylate-butyl acrylate), GO (methacrylated graphene oxide), and PCL (poly(ε-caprolactone)). * *p* < 0.05, ** *p* < 0.01, # *p* < 0.05 compared to the original length, ## *p* < 0.01, *n* = 3.

The Live/Dead assay results showed that the samples were cytocompatible when cultured with C3H10T1/2 cells during triple-shape recovery (Figures 7 and 8). Few dead cells (red dots) were observed in any of the groups, except for the dead control. All other groups showed a cell viability over 95%, with the exception of the pure *t*BA-BA (87% cell viability) and PDMS (92%) groups, indicating the high cytocompatibility of the triple-SMP samples. The pure *t*BA-BA samples showed poor cell attachment, which might have been due to the partial prerecovery of pure *t*BA-BA samples at 30 °C, which contrasts with the stability of the PCL-embedded samples that showed little length change during incubation at 30 °C (Figure 9).

Successful triple-shape-memory behavior under cytocompatible conditions was demonstrated by the samples that contained GO, which showed statistically significant contractile length changes following both thermal and light triggering events. When samples were recovered via thermal triggering followed by light triggering (Figure 9), after incubating at 37 °C for 24h, all samples containing PCL showed a large and significant length change (18.1 ± 2.6% for the 0% GO group, 25.1 ± 1.4% for the 0.3% GO group, and 18.0 ± 4.3% for the 2% GO group, or 20.4 ± 4.2% for the average of the three groups), while pure *t*BA-BA prematurely recovered at 30 °C. After exposure to light with an intensity of 8.69 mW/mm^2^, all samples containing GO showed a smaller but still significant shape change (4.2 ± 0.3% for the 0.3% GO group and 2.8 ± 0.3% for the 2% GO group, or 3.5 ± 0.8% for the average of the two groups) after light recovery, while samples with no GO showed no significant change in length upon exposure to light. The recovered strains in excess of the 20% strain applied during programming are likely due, at least in part, to molecular level strains being trapped in the PCL fibers during fabrication by electrospinning, an effect we and others have previously observed.^21^

## 4 Discussion

The results demonstrate a cytocompatible triple-SMP that can undergo two different shape changes via two distinct cytocompatible triggers—light and heat—during cell culture. Cells cultured on the triple-SMP materials maintained viability greater than 95%. The cytocompatible triple-SMP behavior was achieved via two material components, a *t*BA-BA network that can be triggered near body temperature and electrospun PCL fiber mats with GO that can respond to visible light.

The material characterization results provided additional insight into shape-memory mechanisms. For the thermal trigger, most of the strain recovery was due to the *t*BA-BA. For the light trigger, GO transferred light into heat via a photothermal effect that warmed the surrounding material, causing the melting of PCL. For example, after exposure to light for 5 min, the PCL fibers with GO became coarser (Figure 6), indicative of melting. Meanwhile, no significant difference was found between as-spun fibers and 37 °C treated samples, whether they contained GO or not, indicating that PCL fibers with or without GO did not melt during the thermally triggered recovery. Furthermore, the observed melting of the PCL suggests that exposing the GO-containing samples to longer duration or higher intensity light could produce full light-triggered recovery due to the PCL *T*_m_ being higher than the *t*BA-BA *T*_g_. In addition, it is interesting to note that additional insight into the thermal interplay between PCL and *t*BA-BA could likely be revealed in the future with modeling. Light absorption by GO embedded in PCL is expected to initially and rapidly heat (localization of heat source and short distances for heat diffusion) and melt the PCL to allow recovery of strain fixed by PCL. Over time, this heat will spread but slowly due to the low thermal conductivity of polymers and the larger special dimension of the matrix phase. Should the source of heat from absorption of light persist (with light on), over time, it is expected that the *T*_g_ of the matrix could be exceeded. In practical applications, the times are apparently well enough separated to allow distinct triggering events, one triggered by light and the other by surrounding environment heat.

The results further revealed that the *t*BA-BA component produced more strain recovery than did the PCL component. This may be due to the relatively low weight percentage of PCL—only 8% of the total sample by weight, as the slightly lower than expected value could be due to swelling of the fibrous web by the monomer. In addition, when below 40 °C, there is a substantial difference in the storage moduli of the *t*BA-BA^44^ substrate and the PCL fiber mats,^8^ as we have reported previously, with *t*BA-BA having a storage modulus above 1000 MPa and PCL having a modulus between 10 and 100 MPa. In contrast, both storage moduli drop below 10 MPa when they are above 50 °C. Thus, the strain recovered by the PCL component was not expected to be as significant as that of the *t*BA-BA component. Moreover, the interaction of the independent shape-memory responses of the two components is likely complex, with not only relative compositions and storage moduli affecting the outcome but also the different architectures of the fiber mat and encasing matrix. For example, in prior work employing dual-jet electrospun fiber composites comprising a blended fiber mat that used an enzymatic rather than thermal trigger,^8,9^ we found that, as enzymatically labile shape-fixing fibers were degraded, enzymatically stabile shape-memory fibers produced greater shape change when randomly oriented than when aligned. This prior work thus demonstrated a pronounced role for fiber geometry, with the potential for fiber deformation to deviate from macroscopic behavior. In contrast, given the relative moduli and geometry of the present work in which PCL is a web conformally encased inside pores in an acrylate matrix, we consider it highly likely that during any deformation, the surfaces of those acrylate pores are describing the envelope of strains of all the PCL fibers, limiting the fibers to affine deformation consistent with the macroscopic deformation of the acrylate matrix.

In considering the potential use of triple-SMPs in biomedical applications, one potential limitation of this first triple-SMP is the low strain magnitude demonstrated upon triggering by light, measured in this work at approximately 1.5% (Figure 5C, *t*BA-BA with PCL fibers with 0.3% GO) and 6% (Figure 5D, *t*BA-BA with PCL fibers with 2% GO) or 3.5% strain recovery (Figure 9, light triggering during active cell culture, shorter duration as compared to DMA). This limitation is an addressable one, as the magnitude of strain recovery could be increased by increasing the duration of photothermal triggering, by electrospinning denser PCL fiber mats to increase the weight percentage of PCL, or by improving the mechanical properties of PCL, for instance, by reinforcing with cellulose nanofillers.^45^ Due to the nature of the light guide, a limitation that was observed was that the light guide could only illuminate roughly 60% of the gauge length of the SMP. This equates to roughly 4 mm of gauge length being illuminated. Thus, the calculated values are an underestimate of the actual recovery, with the actual recovery likely being closer to 2.5 and 10% rather than the calculated values of 1.5 and 6%, respectively.

Although cell attachment on the triple-SMP materials was robust, it was observed that cells showed relatively poor cell attachment on the pure *t*BA-BA control samples. The relatively poor cell attachment of pure *t*BA-BA samples (Figure 7) might have been due to the prerecovery of pure *t*BA-BA samples at 30 °C (Figure 9), recovery that could have begun before the cells had fully attached to the substrate.^46,47^ Regardless, cell attachment on the triple-SMP materials—those containing PCL and GO—was robust.

## 5 Conclusions

Here, a triple-SMP that can be triggered under cytocompatible conditions has been successfully developed by encasing a PCL fiber mat in a matrix of *t*BA-BA. The *t*BA-BA network can be triggered by heating to body temperature, and the PCL fiber mat with GO can be triggered by visible light. Both triggering events were found to be cytocompatible. Material characterization provided insight into the distinct shape-memory mechanisms involved. The demonstration of the cytocompatible triple-SMP that can undergo two different shape changes via two distinct cytocompatible triggers—heat and light—during active cell culture can be anticipated to enable the incorporation of triple-shape memory into biomedical devices and strategies.

## Data Availability

The data that support the findings of this study are available from the corresponding author upon reasonable request.

## Supporting Information Available

Three-dimensional printing for sample preparation; overview of the approach wherein tandem triggering is achieved via a photothermally triggered component; diameter of PCL fibers and distribution of GO particles; transition temperature as a function of composition; stepwise shape recovery of the samples; temperature of the material after being exposed to visible light; Fourier transform infrared spectroscopy confirming the methacrylation of graphene oxide; representative traces from dynamic mechanical analysis; representative photograph showing programming of a sample; representative photographs showing the stepwise shape recovery of samples (PDF)
