# Supplementary material for: Thermally and Photothermally Triggered Cytocompatible Triple-Shape-Memory Polymer Based on a Graphene Oxide-Containing Poly(ε-caprolactone) and Acrylate Composite
Source: ACS Appl Mater Interfaces. 2023 Oct 30;15(44):50962–72. doi: 10.1021/acsami.3c13584 (PMC10636728; doi:10.1021/acsami.3c13584)
Supplement: Supplementary file 1 — am3c13584_si_001.pdf [file am3c13584_si_001.pdf]

Supporting Information

A Thermally and Photothermally Triggered Cytocompatible Triple-Shape-Memory Polymer Based on a Graphene Oxide-Containing Poly(ε-Caprolactone) and Acrylate Composite

Junjiang Chen[a,b], Shiyang Sun[a,b], Mark M. Macios[a,b], Elizabeth Oguntade[a,b], Ameya R. Narkar[a,b], Patrick T. Mather[c], James H. Henderson*[a,b]

a BioInspired Syracuse: Institute for Material and Living Systems, Syracuse University, Syracuse, NY, 13244

b Department of Biomedical and Chemical Engineering, Syracuse University, Syracuse, NY, 13244

c Department of Chemical Engineering, Penn State University, University Park, PA, 16802

*Corresponding author:

James H. Henderson, Department of Biomedical and Chemical Engineering, Syracuse University, Syracuse, NY, 13244, Email: jhhender@syr.edu

1. **Three-dimensional (3D) printing for sample preparation**

A spool of commercially available polylactic acid (PLA) filament (Creality, Shenzhen, China) was used for printing. PLA is a recognized semi-crystalline polymer that can be processed using fused deposition modeling (FDM) 3D printing. To print the filament into desired angle bracket molds (dimensions: 20 mm x 20 mm x 4.6 mm) for programming the SMPs, the molds were created in computer aided design software and STL files were converted to g-code using a slicing software (Ultimaker Cura, Utrecht, The Netherlands). Next, the PLA filament was loaded into a 3D printer (Ender 3 Pro, Creality) with a 0.4 mm nozzle diameter for depositing the semi-molten material onto the build plate in a layer-by-layer process. To ensure proper sample adhesion to the build plate, the build plate was covered with Kapton tape and maintained at 50°C. The molds were printed with a nozzle temperature of 220°C, print speed of 30 mm/s, and print flow rate of 125%.

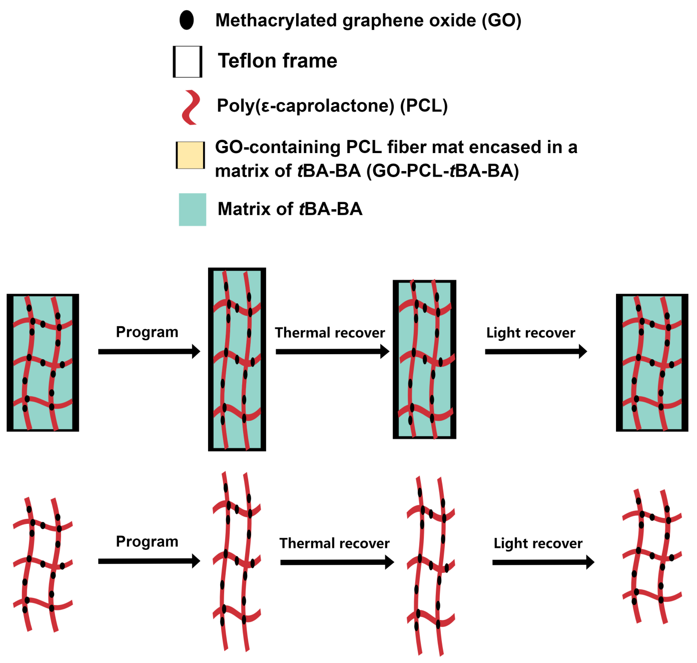

Scheme S1. Overview of approach wherein tandem triggering was achieved via a photothermally triggered component, comprising poly(ε-caprolactone) (PCL) fibers with graphene oxide particles physically attached, embedded in a thermally triggered component, comprising a *tert*-butyl acrylate-butyl acrylate (*t*BA-BA) matrix.

Table S1. Diameter of PCL fibers and distribution of GO particles

0% GO-PCL		0.3% GO-PCL		2% GO-PCL	
diameter (μm)	particles/μm^2	diameter (μm)	particles/μm^2	diameter (μm)	particles/μm^2
1.90 ± 0.63	0	1.92 ± 0.55	0.006 ± 0.002	2.06 ± 0.073	0.025 ± 0.008

Table S2. Transition temperature as a function of composition

Composition	T*g*
Pure *t*BA-BA	37.5 ± 0.3 ˚C
0% GO-PCL - *t*BA-BA	36.1 ± 0.2 ˚C
0.3% GO-PCL - *t*BA-BA	44.6 ± 1.2 ˚C
2% GO-PCL - *t*BA-BA	44.0 ± 0.7 ˚C

Table S3. Stepwise shape recovery of the samples after heating followed by exposure to visible light.

	Shape fixing ratio	Shape recovered after heating	Shape recovered after exposure to light	Total shape recovered
Pure *t*BA-BA	97.61 ± 0.66%	90.85 ± 2.80%	2.45 ± 1.96%	93.30 ± 1.36%
0% GO-PCL- *t*BA-BA	97.46 ± 0.74%	81.77 ± 6.43%	1.36 ± 0.37%	83.13 ± 6.09%
0.3% GO-PCL- *t*BA-BA	98.38 ± 1.42%	58.71 ± 16.5%	11.74 ± 4.25%	70.45 ± 15.82%
2% GO-PCL- *t*BA-BA	98.17 ± 0.90%	46.73 ± 8.51%	29.76 ± 20.14%	76.49 ± 13.45%

Table S4. Temperature of the material after being exposed to visible light with an intensity of 8.69 mW/mm^2 for 5 min.

	Temperature after exposure
Pure *t*BA-BA	35.2 ± 1.9 ˚C
0% GO-PCL- *t*BA-BA	34.5 ± 1.7 ˚C
0.3% GO-PCL- *t*BA-BA	47.0 ± 2.4 ˚C
2% GO-PCL- *t*BA-BA	53.8 ± 3.2 ˚C

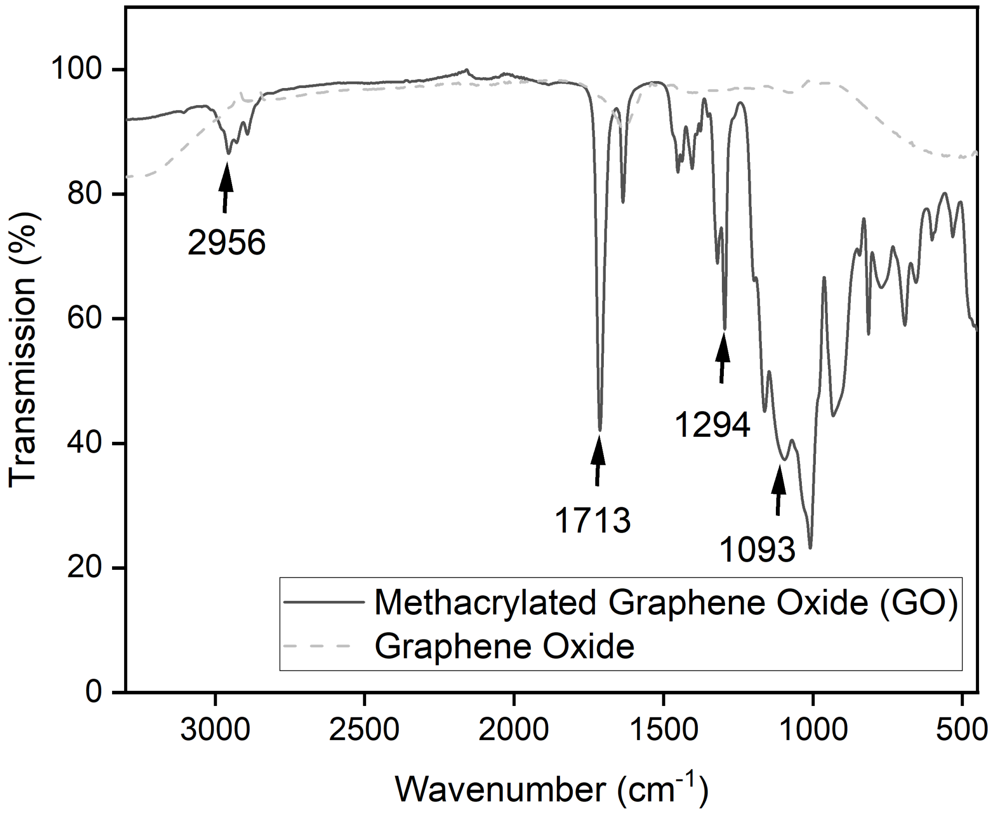

Figure S1. Fourier-transform infrared spectroscopy (FTIR) confirmed the methacrylation of graphene oxide (GO). Arrows point to the following characteristic peaks in decreasing order of wavenumber: 2956 cm^{-1}, C-H; 1713 cm^{-1}, C=O; 1294 cm^{-1}, Si-C; 1093 cm^{-1}, Si-O. GO (methacrylated graphene oxide).

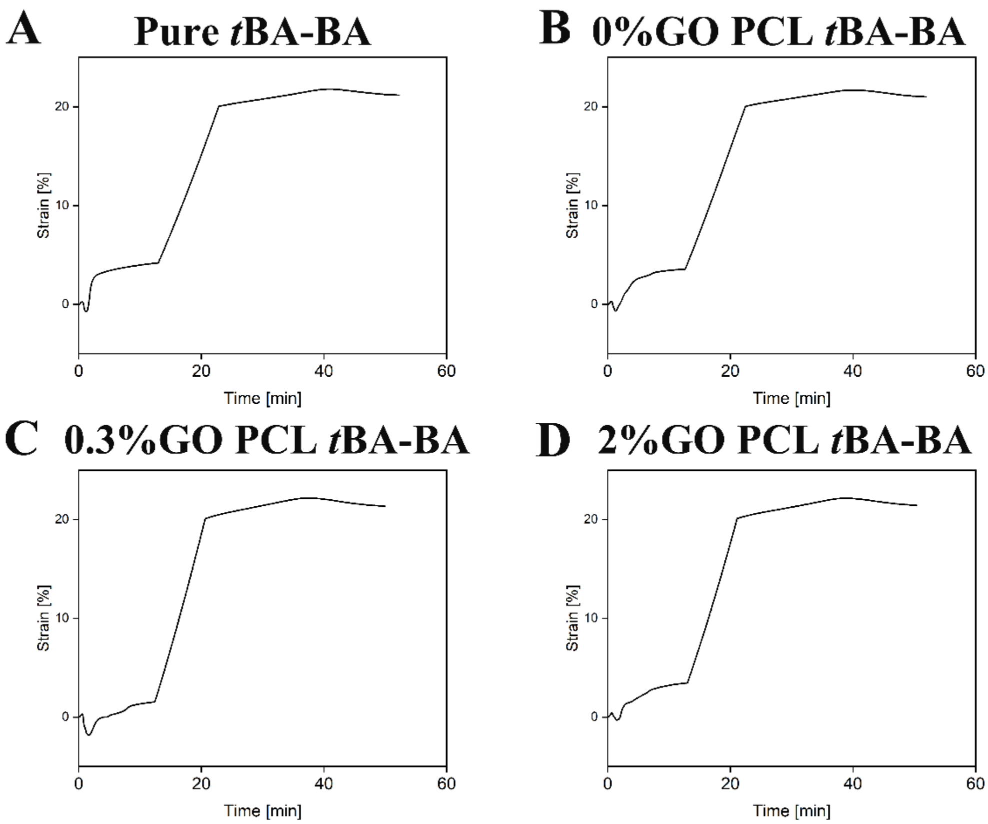

Figure S2. Representative traces from dynamic mechanical analysis (DMA) confirmed that all samples were successfully thermally programmed to 20% strain before light recovery. (A) pure *t*BA-BA, (B) *t*BA-BA with PCL fibers with 0% GO, (C) *t*BA-BA with PCL fibers with 0.3% GO, and (D) *t*BA-BA with PCL fibers with 2% GO. *t*BA-BA (*tert*-butyl acrylate-butyl acrylate), GO (methacrylated graphene oxide), PCL (poly(ε-caprolactone)).

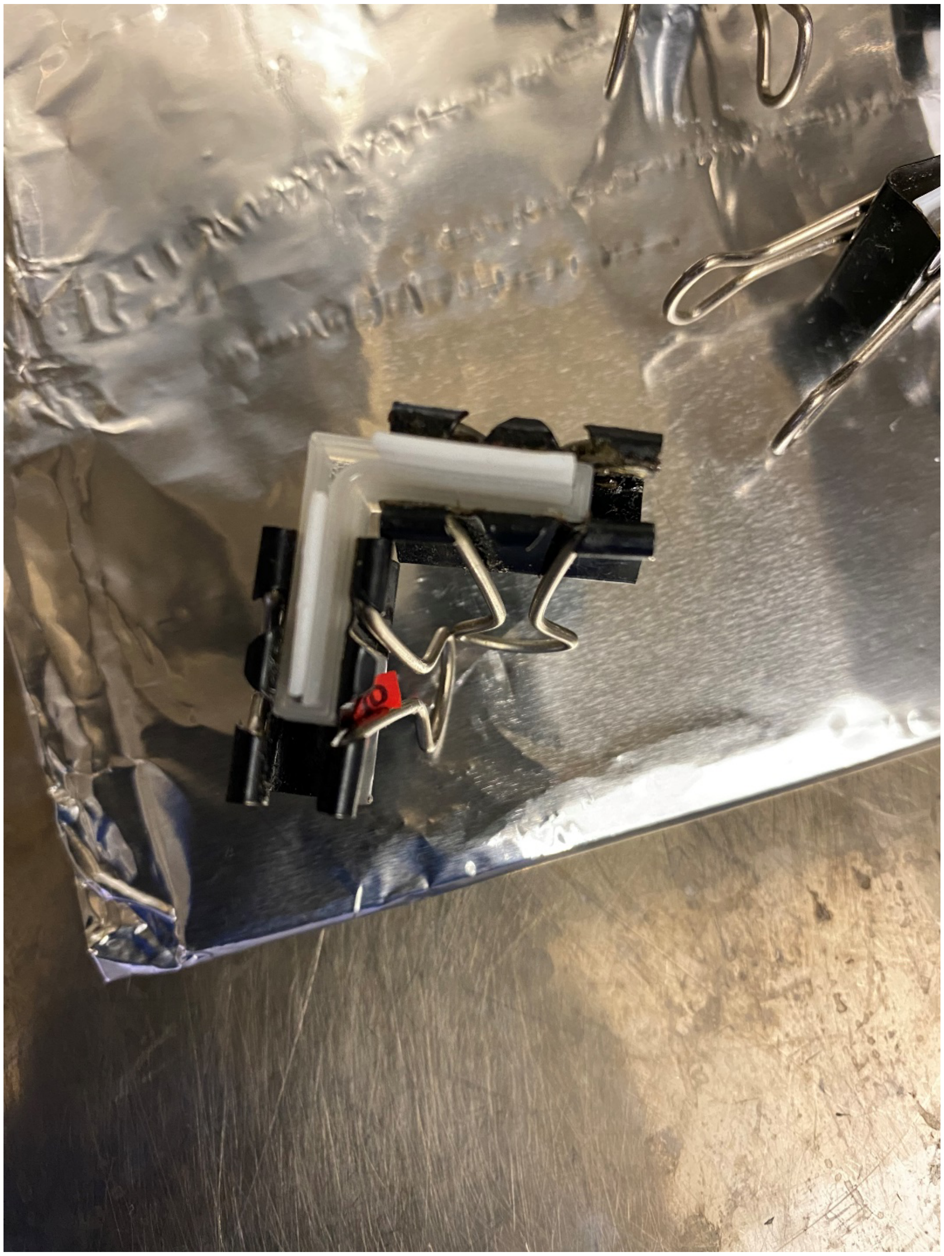

Figure S3. Representative photograph showing programming of a sample into the temporary shape by heating at 55 °C for 5 min and then manually bending to a 90° angle in a bracket mold.

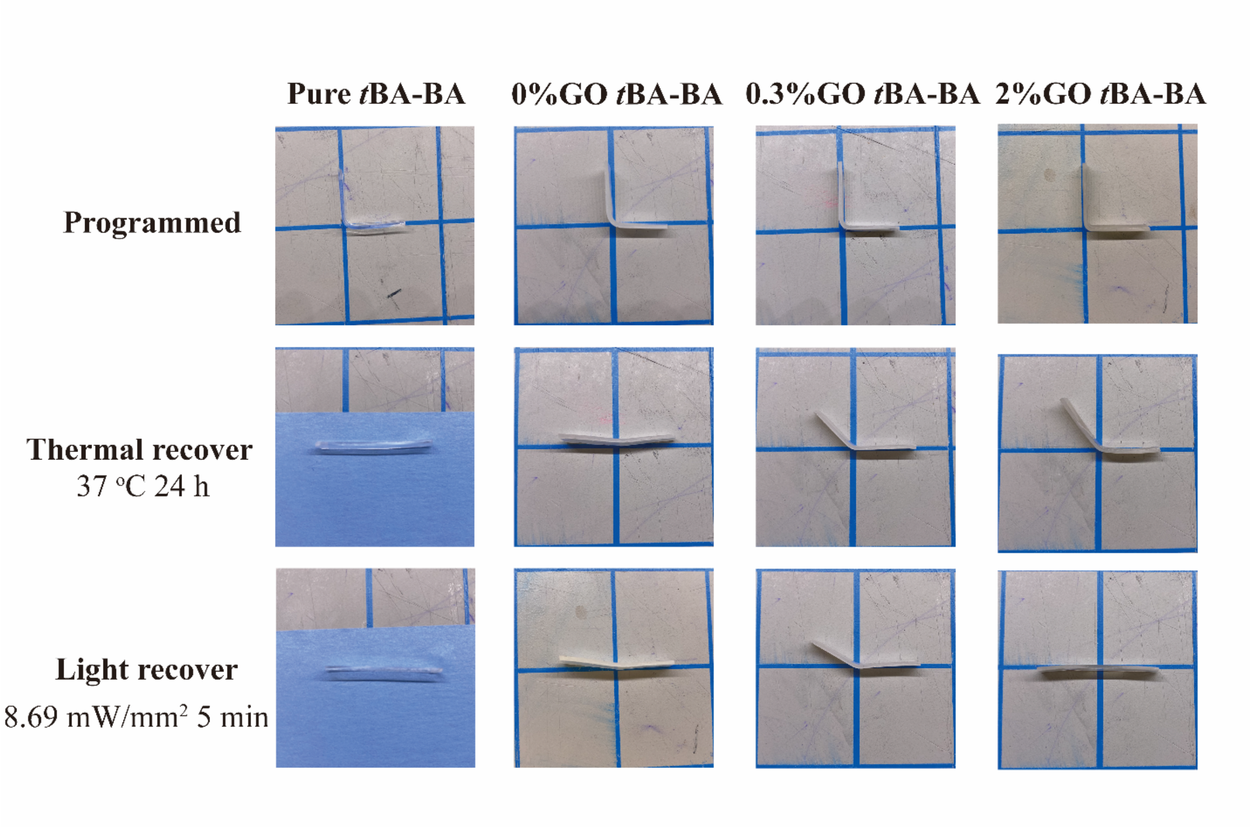

Figure S4. Representative photographs showing stepwise shape recovery of samples after heating followed by exposure to light. The temporary shape was programmed by first heating the samples up to 55 °C and then manually bending to a 90° angle in a bracket mold and fixing at -20 °C. For stepwise shape recovery, samples were first heated to 37 °C in PBS for 24 h, followed by exposure to visible light with an intensity of 8.69 mW/mm^2 for 5 min. *t*BA-BA (*tert*-butyl acrylate-butyl acrylate), GO (methacrylated graphene oxide), PCL (poly(ε-caprolactone)).
